# Moxibustion therapy on lumbar disc herniation: An evidence-based clinical practice guideline

**DOI:** 10.1097/MD.0000000000024347

**Published:** 2021-03-05

**Authors:** Fanghui Hua, Jun Xiong, Haifeng Zhang, Jie Xiang, Shouqiang Huang

**Affiliations:** a Jiangxi University of Traditional Chinese Medicine, Nanchang; b Affiliated Hospital of Jiangxi University of Traditional Chinese Medicine, Nanchang, P.R. China.

**Keywords:** guideline, lumbar disc herniation, moxibustion

## Abstract

**Background:**

Lumbar disc herniation (LDH), as a disease with great disturbance to life and work, is known as the origin of the severe and disabling forms of nerve root pain. Recognized as an increasingly widely accepted treatment, the efficacy of moxibustion on LDH has been affirmed. However, clinical practice guidelines (CPG) for the treatment of LDH with moxibustion have not been developed. Therefore, we will carry out this work following the accepted methodological quality standards.

**Methods:**

The new CPG will be developed according to the Institute of Medicine (IOM), the Appraisal of Guidelines for Research & Evaluation II (AGREE II) and WHO guideline handbook. And then determine recommendations based on high-level evidence. We will set up a Guideline Working Group and define clinical issues according to the PICO principles (Population, Intervention, Comparison, Outcomes). After evidence syntheses and several rounds of Delphi process, we will reach the consensus. In making the guideline, Patient values or preferences, results of peer review, and interest statements are all within the bounds of what we must consider.

**Results:**

As the study is not yet complete, no results can be reported.

**Conclusion:**

So far, we will develop the first CPG for moxibustion of LDH strictly based on systematic methodologies in China. This CPG will establish the standard of LDH in moxibustion therapy.

**Registration number:**

IPGRP-2020CN034.

## 1 Introduction

### 1.1 Description of the condition

Lumbar disc herniation (LDH) is a frequently occurring pathological condition and common spine disease in patients in their 30s to 50s.^[1,2]^ The most prominent symptom is low back pain.^[3]^ It is an adverse threat for patients to their physical function or ability to work, which has a significant impact on national health care spending.^[4]^ About 70% of adults will have low back pain in their lifetime, and 15% to 45% of those affected will fall victim to chronic pain.^[5]^ In China, an epidemiological study on LDH showed that the prevalence of LDH in all kinds of the population was 7.62%.^[6]^ In the United States, the prevalence of lower back pain in adults is 10% to 30% annually, and the lifetime prevalence is 65% to 80%.^[1]^

It is mainly treated surgically or with conventional modalities in western medicine, which includes oral and injection of western drugs, NSAIDs, corticosteroids and anaesthetic drugs.^[7]^ However, these drugs can usually only relieve early pain,^[8]^ and long-term use also brings some side effects, such as hepatorenal toxicity and gastrointestinal reactions.^[9]^ Although surgery results in faster symptom relief, patients undergoing surgery still have to bear the risk of recurrence and complications, such as nerve damage, infection, and hematoma.^[10,11]^

Due to the inadequacy of the above therapies, a alternative therapy is urgently needed to supplement them.

### 1.2 Description of the intervention

As an essential component of traditional Chinese medicine, moxibustion therapy has been widely recommended in clinical practice to treat LDH. Many studies show that moxibustion is superior to western medicine, with a low rate of complications and adverse events. Besides, the costs of moxibustion therapy are within the patient's reach.^[12]^ Moxibustion can regulate the immune function, and the warming effect can enhance the phagocytosis of cells, improve the blood circulation, reduce the excitability of nerves, and eliminate the inflammation of nerves.^[13]^

At present, the most frequently used acupoints in clinical practice are ”Yaoyangguan” (DU 3), ”ashi acupoints,” ”Guanyuanshu” (BL 26), and ”Weizhong” (BL 40).^[14]^ After our initial literature search, we found that more than 280 studies met the inclusion criteria, which was enough to support our CPG. It is proven that moxibustion has efficacy and safety on LDH in accordance with the increasing number of SRs and meta-analysis recently. High-quality SRs or meta-analysis is used as the basis for evaluating clinical efficacy and developing the clinical guidelines.

### 1.3 Description of the objectives

We aim to develop a protocol about a clinical practice guideline (CPG) for the treatment of LDH using moxibustion. The CPG will establish the standard of LDH in moxibustion therapy.

## 2 Methods

### 2.1 Principle

We follow the World Health Organization guidelines,^[15]^ GRADE system,^[16]^ AGREE II instrument,^[17]^ and adhere strictly to the new guideline definition from the IOM.^[18]^ The new CPG will be developed according to the Institute of Medicine (IOM), the Appraisal of Guidelines for Research and Evaluation II (AGREE II) and WHO guideline handbook. Currently registered CPG can be found on the international practice guide registration platform^[19]^ with the registration number IPGRP-2020CN034 (http://www.guidelines-registry.org/guid/878).

### 2.2 Participating institutions, end-users and target population

We launched the guideline at Jiangxi University of Traditional Chinese Medicine. The title will be named, “Moxibustion Therapy on Lumbar Disc Herniation: An Evidence-based Clinical Practice Guideline.” The guidelines will be tailored for use by acupuncturists, physicians, and journal editors. The target population is made up of patients with LDH, and who can accept moxibustion therapy. This guideline covers how to choose the appropriate moxibustion, safety, and efficacy of moxibustion treatment in clinical application.

### 2.3 Guideline working group

We will establish the Guideline Working Group in November 2020, which is made up of three groups as follows: the Guideline Development Group, the Guideline Steering Group and the Guideline Secretary Group. To make gender and geographical representation, 20 members will be recruited through the Guidelines Development Group, who are from the following areas of expertise: 11 acupuncturists (specialise in LDH), 2 traditional Chinese medicine (TCM) physicians, 2 medical clinicians, 2 physiotherapists, a nurse,1 editor, 1 health economist doctor. This Group needs to finish the following:

1. to clarify the guidelines’ scope and draw up Population, Intervention, Comparison and Outcomes (PICOs);
2. to assess the quality of RCTs;
3. to put forward an elementary proposal;
4. to finish a draft guideline and
5. to promote the guideline.

There will be 8 members in the Guidelines Steering Group. It has 3 acupuncturists, 1 expert in evidence-based medicine, 1 TCM physician, 2 physiotherapists and a health economist doctor. This Group's tasks are as follows:

1. to approve the PICOs;
2. to monitor literature retrieval and systematic reviews;
3. to inspect the quality of evidence;
4. to formulate the final recommendations through the revised Delphi technique; and
5. to authorise the guidelines’ release.

There will be 6 members to form the Guideline Secretary Group, which includes 2 acupuncturists, a statistician and 3 experts in evidence-based medicine. The tasks of the Guideline Secretary Group are as follows:

1. to put a literature retrieval and systematic reviews into effect and
2. to inquire patients’ values and tendency.

### 2.4 Declaration of interests and funding support

All the above members will be required to complete a form, which declares conflicts of interest on it, to identify conflicts of interest in potential. This work was supported by the National Natural Science Foundation of China (82060893, 81573835) and “One Thousand Talents Plan” for Introducing and Training High-level Innovative and Entrepreneurial Talents in Jiangxi Province—the first batch of Training High-level Scientific and Technological Innovative Talents (Youth) Project (JXSQ2019201104).

### 2.5 Identifying questions and selecting outcomes

The Population, Intervention, Comparison, Outcomes (PICOs) will be finalized after the determination about the guidelines’ scope by the Guideline Development Group and then approval by the Guidelines Steering Group. The selection of clinical outcomes is the responsibility of the Guidelines Development Group, which also needs to classify them by consensus according to their consequences. We will grade the results on a scale of 1 to 9, with 7 to 9 being critical, 4 to 6 important, and 1 to 3 unimportant.^[16]^ Then, clinical questions will be formulated according to the PICOs principles.

For example: Whether patients with LDH can be treated with moxibustion?

P: all patients with LDH
I: patients who accept moxibustion
C: patients who not accept moxibustion
O: Total effective rate, Oswestry dysfunction index(ODI), the Japanese orthopaedics association score(JOA), Oswestry dysfunction index(ODI), Mac Nab efficacy evaluation criteria, recurrence rate, safety indicators include the occurrence of adverse reactions, improvement of pain, complication, McCormick-score, Prollo-score, Frankel-score.

Which treatment effect is better, moxibustion or non-moxibustion?

P: all patients with LDH
I: Patients treated with moxibustion alone
C: Patients treated with non-moxibustion
O: Effective rate

Is there much difference between moxibustion therapy and moxibustion combined with other therapies?

P: all patients with LDH
I: Patients treated with moxibustion alone
C: Patients treated with moxibustion combined with other therapies
O: Effective rate

### 2.6 Evidence retrieval and synthesis

#### 2.6.1 Databases

The literature search will be conducted systematically until August 30, 2020, in the following seven databases: PubMed, Embase, Cochrane library, SinoMed, CNKI (China), Wanfang (China), and VIP (China).

#### 2.6.2 Search terms

We will take a combined search by using both free words and MeSH items, “moxibustion” and “lumbar disc herniation,” which could balance search specificity and sensitivity. The following search terms will be taken: (lumbar disc herniation or herniated lumbar discs) AND (moxibustion or indirect moxibustion or suspended moxibustion, or direct moxibustion or mild moxibustion or heat-sensitive moxibustion). To ensure the comprehensiveness of the search, we will consult an expert, who is professional in evidence-based medicine, to develop and confirm an proper search strategy. Then, we will carry out a preliminary test according to this strategy to verify the stability of it. Publications in any language will be included in the search

#### 2.6.3 Pilot search

The authors of the systematic reviews update will make a pre-test before the retrieval officially starts to ensure the comprehensiveness and repeatability of literature retrieval. After this text, the authors will discuss the inconsistencies and understand more precisely what the inclusion and exclusion standards about.

#### 2.6.4 Literature selection

We will confirm the literature of initial retrieval, of which duplicate articles will be excluded. First, excluding unrelated studies by reviewing titles and abstracts, then we will read the full text if there are studies that cannot be identified. Finally, studies such as randomized controlled trials, systematic reviews, case–control studies, and meta-analyses will be selected. All numbers of the Guideline Secretary Group, who will be divided into three groups, are responsible for literature selection.

#### 2.6.5 Evidence syntheses

We will adopt systematic reviews without consideration, which followed PRISMA^[20]^ and published during the last 3 years. But if some high-quality systematic reviews have published more than 3 years, we will renew them. If that systematic reviews are of low quality or have not been released, we will use currently available evidence to conducted new systematic reviews.

#### 2.6.6 Evidence assessment

The assessment will be made based on the GRADE tool. It is divided into high, moderate, low, or very low. Then evidence from each study will be assessed on the grounds of outcomes. The quality assessment of the evidence is undertaken by the expert on the guidelines methodology, of which results are then sent to the Guideline Working Group.

### 2.7 Patients’ values and preferences

We consulted patients with LDH to inquire their views on moxibustion and whether they are willing to accept moxibustion treatment. The answers will be considered in the formulation of recommendations. In this study, we will conduct a questionnaire about patients’ value and preference. We will collect and confirm the patient's basic information, including age, job, current address, literacy rate, family income, past medical history, etc, which may influence the patient's choice. Before the investigation formally begins, we will conduct a preliminary test to determine whether the questionnaire is reasonable and feasible. Since the questionnaire will involve some professional medical knowledge, we will conduct relevant training for patients, to complete the questionnaire more accurately. All matters are subject to the patient's voluntary informed consent.

### 2.8 Developing recommendations

After evaluation of evidence quality by GRADE tool, the Guideline Development Group will propound a preliminary proposal based on the result of assessment, the weighting of the merits and demerits as well as the patients’ values and preferences.

After 2 to 3 rounds of Delphi process,^[21]^ repeatedly consulting, summarising and amending, the Guideline Development Group will submit the final opinion as a draft proposal to the Guideline Steering Group, and finally obtained the approval of release. We will reach a consensus concerning the GRADE Grid tool.^[22]^ Each draft of the questionnaire has five options: “strong recommendation,” “weak recommendation,” “unclear recommendation,” “weak recommendation,” and “strong recommendation.” Supposing that over 50% of the experts vote for any option other than “unclear” or over 70% for either options on the same side, we will reach consensus on a recommendation. Supposing that over 50% of the experts support any option other than “unclear,” or over 70% support either option on the same side, we will agree on the recommendations. Except for the above, we shall determine there are different views on the item and need another Delphi process to resolve the dispute.

### 2.9 Peer review

Upon completion, we will refer the guideline to external experts for peer review, and then the Guideline Development Group collects and documents recommendations and review process. The merits of recommendations suggested by experts will be evaluated by us together.

### 2.10 Publishing of the guideline

To improve the integrity of guidelines and the quality of reporting, we will report the guideline correctly in the format suggested in the RIGHT checklist.^[22]^ The publication, which will be translated into English and Chinese, is expected to be existed in the relevant journals in 2021 and updated regularly.

### 2.11 Promotion of the guideline

After publish of this guideline, Jiangxi University of Traditional Chinese Medicine will popularize it in ways as follows:

1. the guideline will be suggested in 3 years at LDH conferences;
2. the Guideline Working Group will publish the research related to guideline;
3. Both Chinese and English versions of the guidelines will be published on the official health website.

And the guideline will be updated in future.

## 3 Discussion

### 3.1 Limitations

Given that moxibustion belongs to the field of TCM, most of the sites included in the study are in China and the application of moxibustion in other countries and regions needs further study. In addition, the fitness of moxibustion among populations in different countries should also be considered.

### 3.2 Contributions

First, as far as we know, this is the first CPG protocol to evaluate moxibustion therapy in LDH patients. Secondly, the results of this practice guideline protocol will assist in the selection of the best treatment for LDH in clinical decision-making. Lastly, the results are helpful for the formulation of the best moxibustion treatment of LDH, including the correct operation method, as well as the relationship between point selection, moxibustion time, moxibustion quantity, and curative effect.

## 4 Conclusion

The formulation of this CPG will help to establish the standard of LDH in moxibustion therapy. The principles and standards of evidence-based medicine will be followed when developing guidelines for the treatment of LDH in moxibustion therapy. It is expected that this CPG will be used by clinicians not only for treating patients with LDH but also for teaching activities.

## Acknowledgments

We are very grateful to the study participants who took the time to participate.

## Author contributions

**Conceptualization:** Haifeng Zhang.

**Data curation:** Jie Xiang, Shouqiang Huang.

**Writing – original draft:** Fanghui Hua.

**Writing – review & editing:** Jun Xiong.

## Corrections

The funding number, 81573835, was originally left out of the article and has since been added.
